# Mother–Child Synchrony and Behavioral Problems in Chinese Children: A Preliminary fNIRS‐Hyperscanning Study

**DOI:** 10.1002/brb3.71414

**Published:** 2026-04-16

**Authors:** Chao Liu, Xi Liang, Xiaoxu Meng, Nanhua Cheng, Shan Lu, Liu Bai, Bingbing Song, Chunming Lu, Zhengyan Wang

**Affiliations:** ^1^ Beijing Key Laboratory of Learning and Cognition, Research Center For Child Development, School of Psychology Capital Normal University Beijing People's Republic of China; ^2^ State Key Laboratory of Cognitive Neuroscience and Learning & IDG/McGovern Institute for Brain Research Beijing Normal University Beijing People's Republic of China

**Keywords:** behavioral problems, behavioral synchrony, fNIRS, mother–child interaction, time‐lagged neural synchrony

## Abstract

**Introduction:**

Parent–child synchrony, defined as the coordination of biological and behavioral processes during social interactions, serves as a critical predictor of children's psychological health and behavioral outcomes. Although recent research has examined its neurobiological underpinnings, the longitudinal relationship between mother–child synchrony and preadolescent behavioral problems in China remains underexplored.

**Methods:**

This longitudinal study followed mother–child dyads at ages 7, 9, and 11, focusing on the trajectory of children's behavioral problems. At age 7, functional near‐infrared spectroscopy was employed during a mother–child drawing task to measure neural synchrony. Neural synchrony was assessed using wavelet transform coherence to capture both timealigned and time‐lagged dynamics during naturalistic mother–child interaction. Group‐level analyses linked these neural patterns to behavioral synchrony and developmental outcomes using regression models and exploratory structural equation modeling.

**Results:**

Results revealed that time‐lagged neural synchrony, involving the mother's temporoparietal junction and the child's dorsolateral prefrontal cortex, was significantly associated with the predominant form of temporally sequential behavioral synchrony. Furthermore, mother‐led child‐following behavioral synchrony and 13‐s time‐lagged neural synchrony were linked to fewer externalizing problems in children.

**Conclusions:**

Main findings reveal that specific patterns of time‐lagged mother–child neural synchrony, particularly when the mother leads and the child follows, are associated with reduced externalizing behavioral problems in children. These findings provide preliminary evidence regarding neurocognitive processes relevant to children's social‐emotional development from middle childhood to preadolescence. These findings are consistent with emerging literature indicating that delayed but structured interbrain dynamics may contribute to co‐regulated emotional development. However, the current evidence remains preliminary and warrants further validation using multimodal synchrony measures and larger longitudinal cohorts to establish causal pathways.

## Graphical abstract

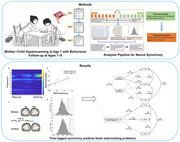

## 1 Introduction

Behavioral problems are common among children (Cui et al. 2021; Xu et al. 2020) and often emerge as early as preadolescence, a critical period of developmental transition from childhood to adolescence. These problems have an immediate impact on children's development and can successfully predict future difficulties such as reduced academic performance (Fu et al. 2016) and long‐term mental health issues (Chen et al. 2012; Reef et al. 2010). Although externalizing problems tend to decline on average after early childhood, substantial individual differences in symptom trajectories emerge during preadolescence, and externalizing behaviors during this stage remain developmentally meaningful, as they reflect stable individual differences and are associated with later adjustment‐related processes (Pinquart 2017; Rothenberg et al. 2020). As such, behavioral problems in preadolescence represent a vital challenge for parenting interventions (Dietz 2020; Moretti et al. 2015). Therefore, the importance of early prediction on these problems has been widely recognized in both academic and clinical fields.

Parent–child synchrony, defined as the coordination of biological and behavioral processes between parent and child during social interactions (Feldman 2007), is considered a critical predictor of children's psychological health and behavioral outcomes (Li et al. 2016; Murray et al. 2014; Shi et al. 2022). Rooted in attachment theory (Bowlby 1969), synchrony during early childhood fosters the development of essential relational skills, including empathy, cooperation, and perspective‐taking (Feldman 2020). Beyond early childhood, increasing synchronous dialogue reflects children's growing capacities for dealing with complex social interactions and the navigation of parent–child conflicts and in enhanced autonomy (Laursen et al. 1998; McCurdy et al. 2020). This developmental shift suggests that the influence of parent–child synchrony on socioemotional adjustment may differ from patterns observed during early childhood (Birk et al. 2022). Accordingly, the function and meaning of parent–child synchrony may differ during middle childhood and the transition into preadolescence, potentially exerting distinct influences on children's behavioral regulation and socioemotional adjustment compared to early childhood (Birk et al. 2022; Harrist and Waugh 2002). Recent studies have begun exploring the neurobiological underpinnings of these dynamics, particularly by focusing on mother–child neural synchrony and its influence on young children's behavioral problems (Quiñones‐Camacho et al. 2020; Quiñones‐Camacho et al. 2022; Su et al. 2024). However, although parent–child neural synchrony has been shown to play a role from early childhood into adulthood (Levy et al. 2017; Piazza et al. 2020), much remains unknown about how neural synchrony during preadolescence specifically shapes children's behavioral problems, particularly from a longitudinal perspective. The present study aims to address this gap by investigating how dyadic behavioral and neural synchrony within the parent–child relationship is associated with the development of behavioral problems from middle childhood to preadolescence.

### 1.1 Parental‐Child Synchrony Definition and Its Measurement

Given the complexity of interpersonal behaviors and physiological responses in parent–child interactions (Bell 2020; Provenzi et al. 2018), it is necessary to conduct a systematic review of the definitions and measurements of behavioral and neural synchrony across different contexts.

#### 1.1.1 Behavioral Synchrony

Parent–child behavioral synchrony usually occurs during interactions between parents and children when they are engaging in reciprocal, contingent, and temporally coordinated behaviors while maintaining a shared focus of attention (Feldman 2007; Harrist and Waugh 2002; Hoyniak et al. 2021). The dyadic behavioral synchrony typically involves two patterns: one is concurrent behavior that occurs simultaneously or within the same epoch (Harrist and Waugh 2002), and the other is a temporally sequential or contingent response pattern within a time window (Feldman 2007; Lunkenheimer et al. 2013). While the former is common in early childhood, the latter tends to be more prominent beyond early childhood (Birk et al. 2022; Brown et al. 2023; Bureau et al. 2021). This developmental shift suggests that sequential behavioral synchrony may become particularly relevant for understanding parent–child coordination and behavioral regulation during middle childhood and preadolescence (Bureau et al. 2021; Birk et al. 2022; Brown et al. 2023; Kerns and Seibert 2021). More importantly, as children enter school and begin to direct activities, they become increasingly adept at forming collaborative relationships with their parents, known as “goal‐directed partnerships” (Kerns and Seibert 2021). Consequently, the pattern of behavioral synchrony shifts from simultaneous behavioral synchrony to temporally sequential synchrony (Birk et al. 2022; Brown et al. 2023). Therefore, in this study, we will observe both simultaneous and temporally sequential behavioral synchrony during a collaborative task in parent–child interactions in line with earlier research (Feldman 2007; Markova et al. 2019; Yarmolovsky and Geva 2024).

#### 1.1.2 Neural Synchrony

On a biological level, neural synchrony between parent and child is emerging as a biomarker of parent–child interaction. Behavioral synchrony provides the foundation for the development of neural synchrony during social interactions (Feldman 2012a, 2017). Neural synchrony, characterized by a reciprocal and dynamic interplay among the neural activity of interacting individuals (Holroyd 2022), involving synchronized oscillations or aligned neural firing patterns (Hasson et al. 2012; Uhlhaas et al. 2009; Wilson and Wilson 2005). Previous studies have used various neuroimaging techniques and analytic methods to characterize the neural synchrony, including wavelet transform coherence (WTC) in fNIRS, phase locking value in EEG, and Pearson correlation in fMRI (Jiang et al. 2021). Due to its affordability and reduced sensitivity to movement artifacts, fNIRS has become the preferred method, especially for studies involving children performing tasks in naturalistic settings (Lloyd‐Fox et al. 2010; Reindl et al. 2018; Hakim et al. 2023). Consequently, we will employ WTC in fNIRS to measure and quantify neural synchrony during parent–child interactions.

Previously, two patterns of neural synchrony have been identified and investigated: time‐aligned and time‐lagged neural synchrony (Jiang et al. 2021). Time‐aligned neural synchrony averages WTC values within the same task block, highlighting immediate brain coupling during interactions (Cui et al. 2012). Time‐lagged neural synchrony, however, captures the temporal sequence of interactions, reflecting directional influences and temporally causal relations between individuals’ cognitive processes (Jiang et al. 2021; Kok et al. 2017; Schilbach and Redcay 2024). In the present study, we will examine both time‐aligned and time‐lagged to better understand mother–child interaction.

#### 1.1.3 The Relations Between Behavioral Synchrony and Neural Synchrony

Time‐aligned neural synchrony occurs when the sensory system of one person couples with the motor system of another, facilitating coordinated behaviors like turn‐taking (Wilson and Wilson 2005). According to the phase reset model, the receiver's neural oscillations adjust to the sender's rhythm, creating a shared timing system (Brandt 1997). In the context of parent–child interactions, neurobehavioral synchrony is thought to enhance learning by regulating the child's needs (Alonso et al. 2024), and neural synchrony can also be externally triggered through joint attention (Leong et al. 2017). A growing body of research has supported the association between time‐aligned neural synchrony and simultaneous behavioral synchrony during early childhood (Hoyniak et al. 2021; Nguyen et al. 2020; Reindl et al. 2022; Liu, Han, et al. 2024).

The predictive coding model suggests that neural activity for predicting current input in one individual precedes the neural response of another individual to the same input (Fischer and Whitney 2014; Kok et al. 2017). This predictive process results in a neural synchrony lag compared to behavioral synchrony, typically by several seconds, which often aligns with the duration of specific interactive behaviors (Jiang et al. 2021). Recent studies support this assumption by demonstrating similar time lags across different contexts. For example, one study found a 4‐s neural synchrony lag aligning with verbal response intervals between adult romantic couples (Long et al. 2022). In a teaching context, a 10‐s neural synchrony lag corresponded to the periods of asking and answering questions between teachers and students (Zheng et al. 2018). These studies involve participants who are relatively older and have already developed advanced social cognitive abilities related to shared intentionality. However, even younger children have demonstrated early forms of this social cognitive ability, as highlighted by Tomasello (2019). In a recent example, a study focusing on child participants examined mother–child shared book reading and identified an 11‐ to 18‐s neural synchrony lag, which aligned with the average 16‐s duration of interaction turns, making it particularly relevant to our study context (Zhai et al. 2023). This suggests that time‐lagged neural synchrony likely reflects the neural basis of turn‐taking and mutual responsiveness during social interactions, capturing how participants adapt to each other's behaviors over time to achieve shared understanding and coordination (Jiang et al. 2021).

### 1.2 The Developmental Framework of Parent–Child Synchrony and Socioemotional Skills

Two frameworks have explored coordinated psychobiological responses within the parent–child dyad (Bell 2020). The first is Feldman's biobehavioral synchrony model, which emphasizes the co‐regulation of behavior and physiology between parent and child in early parent–child interactions. Specifically, the model posits that a parent's responsiveness fosters a child's self‐regulation. Moreover, it suggests that early synchrony, largely parent‐driven, is crucial for the development of brain networks related to social engagement and emotional regulation, supporting socioemotional skills such as empathy, social awareness, and relationship‐building (Feldman 2012a, 2012b).

While Feldman's model focuses on early parent‐driven interactions, the second framework, shared intentionality (Searle 1983), shifts the focus to interpersonal goals, cooperation, and dyadic decision‐making (Fishburn et al. 2018). Unlike Feldman's biobehavioral synchrony model, which centers on early development, shared intentionality examines coordinated behavioral and physiological responses in adult dyads (e.g., Zhang, Yin, et al. 2024). Based on Tomasello's (2019) extension of shared intentionality in the field of social cognitive development, this framework offers a novel perspective on studying synchrony in parent–child interactions, particularly during middle childhood, a developmental stage marking a key step toward becoming reasonable and responsible individuals. Supporting this perspective, Zhao et al. (2021) demonstrated that time‐lagged neural synchrony during mother–child interactions in middle childhood is a significant predictor of children's compliance, a key socialization goal in the Chinese cultural context, often referred to as *tinghua* (听话), meaning obedient or well‐behaved.

### 1.3 Relations Between Parent–Child Synchrony and Behavioral Problems

#### 1.3.1 Behavioral Synchrony and Behavioral Problems

Parent–child synchrony plays a crucial role in fostering attachment between parents and children (Bureau et al. 2014; Feldman 2015; Lindsey and Caldera 2015), thereby serving as a positive indicator of social adjustment across development (Feldman 2010; Feldman et al. 2013). Consistent evidence indicates that higher levels of simultaneous parent–child behavioral synchrony during early childhood are strongly associated with decreased behavioral problems and increased socioemotional competence (Feldman and Eidelman 2004; Feldman 2015; Harrist and Waugh 2002). However, the predictive power of simultaneous parent–child behavioral synchrony for behavioral problems in children beyond the age of three appears to weaken. For example, Quiñones‐Camacho et al. (2022) found that simultaneous mother–child behavioral synchrony during infancy did not predict internalizing or externalizing problems at preschool age.

Autonomy, or the feeling that one's actions align with their own values and interests, is considered crucial from toddlerhood and maintains significance throughout childhood (Deci and Ryan 2000). Parental support for children's autonomy during their school years also positively impacts their emotional well‐being (Cheung et al. 2016; Matte‐Gagné et al. 2015; Sirois et al. 2022). Child‐led mother‐following (CLMF) synchrony, characterized by the child taking the lead in activities and decision‐making within the parent–child relationship, is believed to facilitate the development of autonomy, competence, and relatedness—core components of self‐determination theory (Deci and Ryan 2000; Sobel et al. 2021). Thus, it is reasonable to anticipate that increased CLMF synchrony might be associated with fewer behavioral problems. The self‐determination theory states that the universal desire for autonomy is expressed differently across cultures (Deci and Ryan 2000). Children from various societies may interpret parental behaviors differently (Choe et al. 2023; Soenens and Beyers 2012), perceiving them as either intrusive or caring. In Chinese culture, the concept of *guan* (管) reflects Confucian beliefs about parental duties, encompassing training and includes caring, loving, and governing (Chao 1994). *Guan* involves parents guiding and providing for their children to help them succeed, and children understanding and following their parents’ instructions (Lin 2003; Wu 2013). Within this framework, parental direction is often viewed as an expression of care and responsibility rather than control. This conceptualization underscores the relational and culturally embedded nature of parental leadership within Chinese families, suggesting that interaction patterns such as mother‐led child‐following (MLCF) synchrony may carry different meanings across cultural contexts.

Previous research suggests that sequential synchrony in parent–child interactions is a stronger predictor of behavioral problems at preschool age or during middle childhood (Brown et al. 2023; Bureau et al. 2021; Lunkenheimer et al. 2020; Rothenberg et al. 2020; Serbin et al. 2015). Similarly, research has consistently found a link between self‐reported parental *guan* and better school adjustment (Lan et al. 2019). Based on these findings, we propose that both CLMF and MLCF behavioral synchrony may serve as more powerful predictors of behavioral problems during middle childhood compared to simultaneous parent–child behavioral synchrony, such as joint attention.

#### 1.3.2 Neural Synchrony and Behavioral Problems

Neural synchrony promotes interpersonal bonding and shared mental states through mechanisms such as shared representation and predictive coding between two individuals (Jiang et al. 2021). This optimization of internal models of complex dynamic environments enhances memory and attention toward the interacting partner, reducing cognitive resources needed during social interactions (Dai et al. 2018; Ferrari‐Toniolo et al. 2019; Fischer and Whitney 2014; Jiang et al. 2015; P. Kok et al. 2017; Stephens et al. 2010; Zheng et al. 2018). Evidence shows that shared neural representations developed through parent–child interactions are crucial for children's social adjustment, indicating that prefrontal cortex (PFC) neural synchrony may underlie dyadic attunement, facilitating behavioral synchronization and broader socialization processes (Quiñones‐Camacho et al. 2020, 2022; Reindl et al. 2018; Schilbach and Redcay 2024).

Miller et al. (2019) conducted a study in the United States with preadolescent children and found that stronger parent–child time‐aligned PFC neural synchrony during cooperation was linked to better emotion regulation in both the parent and child. This finding suggests that time‐aligned PFC neural synchrony may serve as a potential mechanism for dyadic attunement. Similarly, recent studies conducted in both the United States and China, encompassing children in early and middle childhood, have demonstrated that reduced time‐aligned PFC neural synchrony during parent–child interaction was associated with potential behavioral problems (Quiñones‐Camacho et al. 2020; Zhang et al. 2024), highlighting the importance of this synchrony in maintaining healthy interactions. Furthermore, Quiñones‐Camacho et al. (2022) showed that time‐aligned neural synchrony during play could predict changes in behavioral problems over time in preschool‐aged children. Collectively, these findings suggest that PFC neural synchrony is critical for dyadic coordination and socialization. While these studies provide valuable insights, the contexts often do not fully capture the complexity of real‐world interactions and the role of predictive processing. Future research could benefit from exploring how predictive processes in parent–child interactions influence children's developmental outcomes, as prediction may further enhance shared neural processes (Zheng et al. 2018).

In real‐life interactions, predictive coding enhances interpersonal interactions by making communication more efficient (L. Liu et al. 2020), reducing cognitive effort (Dai et al. 2018), improving emotional attunement (Long et al. 2021), facilitating learning (Zhao et al. 2021; Zheng et al. 2018), and supporting cooperation (Jiang et al. 2015). This process is related to time‐aligned neural synchrony involving predictive coding of higher cognitive functions. A study conducted in China with middle childhood children found that parent–child time‐lagged temporoparietal junction (TPJ) synchrony during cooperative activities, such as drawing together, is associated with the child's compliance (Zhao et al. 2021). In Chinese teacher and students’ interactions, the teacher's TPJ activity precedes the student's anterior temporal cortex activity by 10 s, indicating the teacher's predictive role (Zheng et al. 2018). In particular, a recent study conducted in China with preschool‐aged children demonstrated that, within an observational learning context, only the time‐lagged neural synchrony in the PFC can track children's cognitive processes of inferring intentions from adult models’ decision‐making behaviors and is associated with children's learning outcomes (Zhao et al. 2022). These interactions involve neural synchrony not only in the PFC but also in the TPJ, facilitating better mutual understanding and prediction of actions (Jiang et al. 2015; Nguyen et al. 2020; Zheng et al. 2018).

The PFC is primarily responsible for planning, decision‐making, and executive control, while the TPJ plays a significant role in understanding others’ intentions and emotions (Casey et al. 2000; Gvirts and Perlmutter 2020; Hoehl et al. 2021; Samson et al. 2004; Saxe and Wexler 2005). The interplay between the PFC and TPJ allows parents and children to better understand and predict each other's actions, thereby improving the efficiency and effectiveness of cooperative tasks (Nguyen et al. 2020; Nguyen et al. 2021). This dynamic is reflected in time‐lagged neural synchrony associated with predictive coding. Such neural synchrony may not only facilitate immediate cooperative behavior but also positively impact children's social cognitive abilities and the long‐term development of their social adjustment.

### 1.4 The Present Study

The present study aimed to investigate the neurobiological mechanisms underlying the long‐term relationship between mother–child synchrony in cooperative interactions and behavioral problems in children from middle childhood (7 years old) to preadolescence (11 years old). To address these issues, mother–child dyads were recruited to perform a cooperative drawing task when the children were 7 years old, and children's behavioral problems were tracked based on maternal reports over the subsequent four years. fNIRS hyperscanning was employed to measure hemoglobin concentration changes in both the mother and the child. Both time‐aligned and time‐lagged neural synchrony were calculated to index the neural responses of both the mother and the child to cooperative drawing. The study is guided by two research questions: (1) What is the relationship between behavioral synchrony and neural synchrony? Specifically, we hypothesize that time‐aligned neural synchrony is related to simultaneous behavioral synchrony (i.e., joint attention), whereas time‐lagged neural synchrony is related to temporally sequential behavioral synchrony. (2) What is the relationship between mother–child synchrony and behavioral problems in children? We anticipate that higher levels of mother–child synchrony, characterized by both behavioral and neural alignment, will correlate with fewer behavioral problems in children. Given that the PFC and the TPJ are key regions of the mutual social attention system, we expect neural synchrony in these regions to be particularly indicative of positive outcomes.

## 2 Materials and Methods

### 2.1 Participants

We initially recruited 48 mother–child dyads through advertisements in Beijing. The mother–child dyads visited our laboratory at three time points: T1 in 2017, T2 in 2019, and T3 in 2021. According to public health research standards (Matthews and Gallo 2011), inclusion criteria for participants included being right‐handed and having normal hearing and vision, with no language, neurological, or psychiatric issues. Written informed consent was obtained from the mothers of the children. Among mothers, 90% had a bachelor's degree or higher (3% high school, 6% college, 51% bachelor's, 40% master's and higher). Families reported a range of monthly incomes: 9% earned less than ¥10,000; 18% earned ¥10,000–¥20,000; 42% earned ¥20,000–¥30,000; 24% earned ¥30,000–¥40,000; and 6% earned over ¥40,000.

From the initial 48 mother–child pairs, eight children declined to wear the optode probes. Following data quality checks and accounting for attrition and other sample exclusions, the final sample at T1 comprised 33 mother–child pairs (18 boys, 15 girls; children's mean age = 7.34, SD = 0.27; mothers’ mean age = 37.85, SD = 3.23). At T2, 17 children (12 boys, 5 girls; mean age = 9.47, SD = 0.19) participated, and at T3, 20 children (14 boys, 6 girls; mean age = 11.12, SD = 0.31) participated. The sample size was similar to previous studies that measured neural synchrony using fNIRS (Liu et al. 2024; Piazza et al. 2020).

### 2.2 Procedure

During each visit, the mother completed a series of questionnaires designed to assess family demographics and child behaviors. At T1, the mother–child dyad engaged in a cooperative drawing task in the laboratory. The following section provides a detailed description of the setting and process.

**FIGURE 1:**
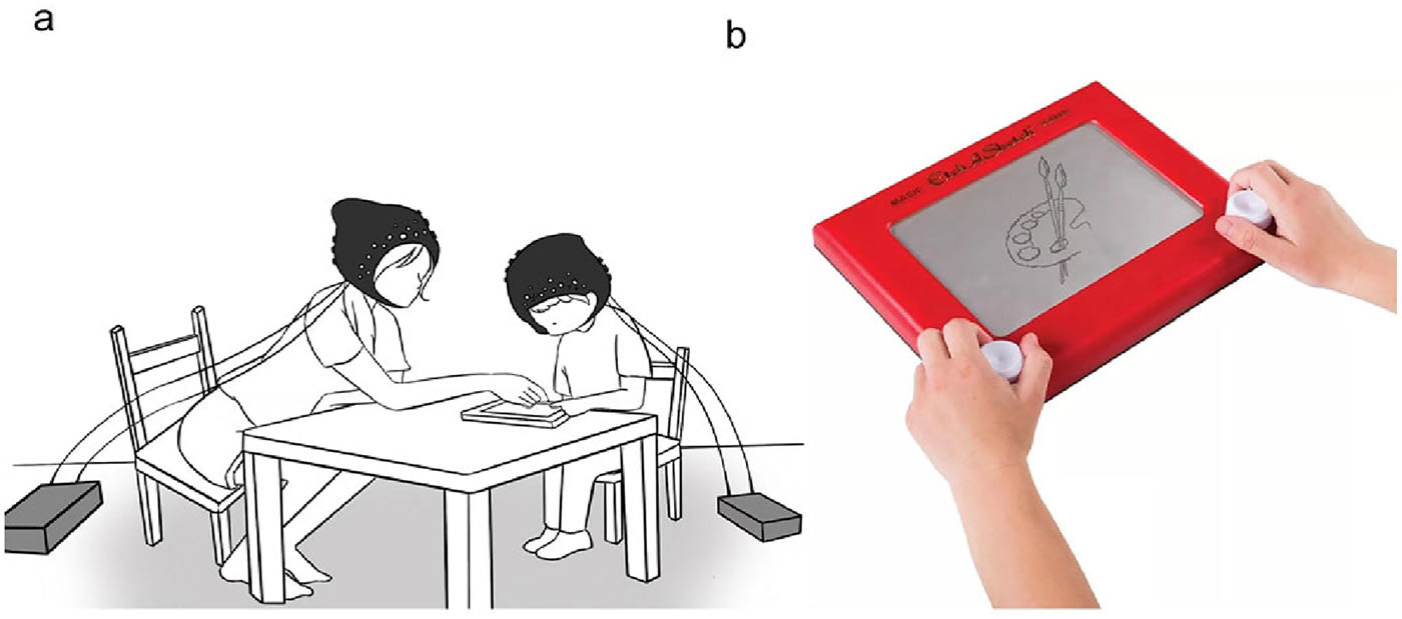
The mother–child drawing task. (a) A child was drawing a picture together with his mother using (b) the drawing board.

The task aimed to measure neural synchrony between the mother and child. fNIRS caps were placed on both the mother and child, and the devices were calibrated for optimal signal quality. The dyad then entered a quiet room for the task. The entire session was video‐recorded to capture their interactions. Both participants were seated at a table positioned at a 90° angle to each other, facilitating comfortable interaction and allowing the camera to capture their faces during the task (Figure 1a).

The session began with a 5‐min resting phase, during which the dyads were instructed to remain still with their eyes closed, relax, and minimize motion (Zhao et al. 2021). Before the 7‐min interactive session, the experimenter introduced the game rules and explained how to use the Etch‐A‐Sketch, a drawing toy with a gray screen and two knobs that create drawings with aluminum powder. One knob controls horizontal lines, while the other controls vertical lines. In the task, the mother and child used their respective Etch‐A‐Sketch knobs to collaboratively draw either a ship or a house within a limited time. They were allowed to communicate both verbally and nonverbally (e.g., through facial expressions, gestures, or touch). While the instructions emphasized the importance of cooperation to complete the drawing, they did not specify whether the mother should lead or assist. The task was framed as a cooperative activity, and participants were not informed that their performance would be evaluated. No formal assessment of task performance or the final drawing was conducted.

At the end of each visit, children were given a token of appreciation. The study protocol was approved by the Institutional Review Board of the Psychological Ethics Committee of Capital Normal University and the State Key Laboratory of Cognitive Neuroscience and Learning at Beijing Normal University.

### 2.3 fNIRS Data Acquisition

**FIGURE 2:**
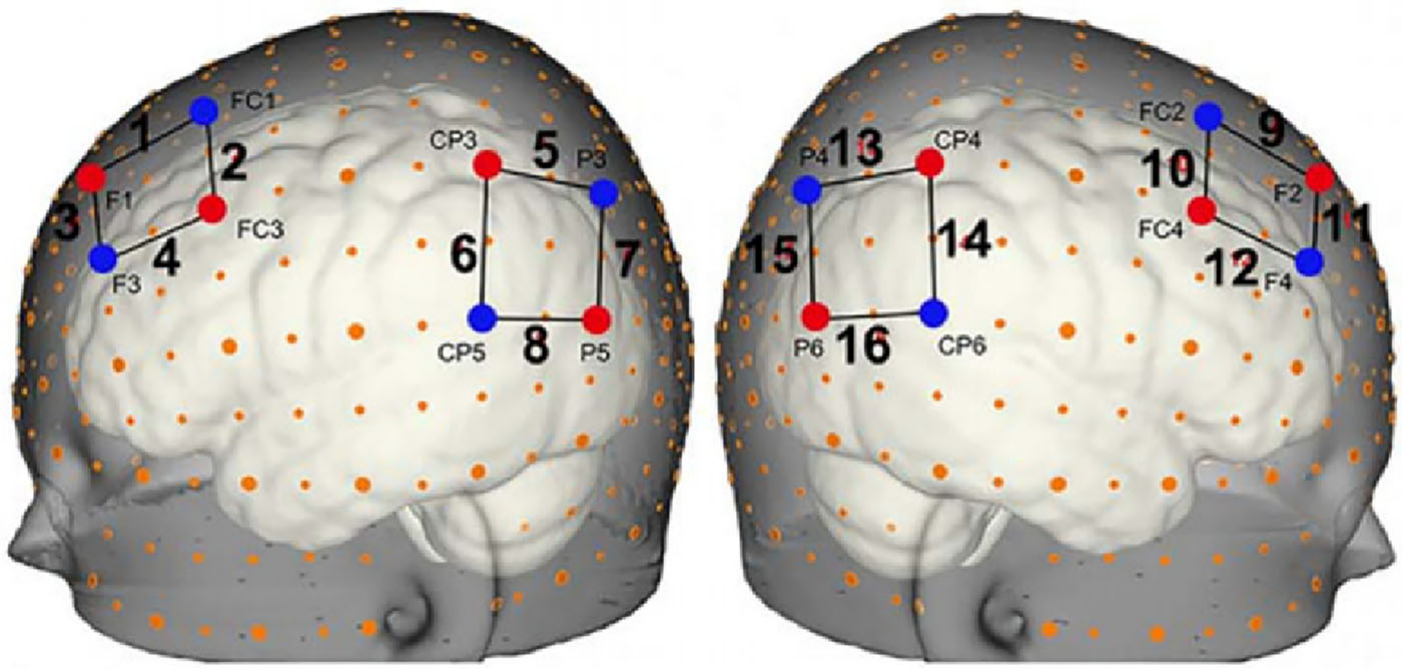
The fNIRS probe set consists of eight sources (red points) and eight detectors (blue points) placed across the left and right hemispheres, resulting in a total of 16 measurement channels. The measurement channels are represented by lines connecting the sources and detectors, with each channel numbered in black. The positions of the sources, detectors, and the measurement channels are referenced to the 10–10 system, as indicated by the markers (orange points) scattered throughout the brain, which show the standard electrode positions.

Two NIRSport optical topography systems (NIRx Medical Technologies, NY, USA) were used to collect raw data, with wavelengths at 760 and 850 nm and a sampling rate of 7.81 Hz. Each participant used four sets of custom‐designed optode probes to cover the PFC and TPJ regions in both the left and right hemispheres (Figure 2). Each probe set included two sources and two detectors, spaced 30 mm apart, resulting in four measurement channels (CHs). The positions of the sources and detectors were mapped according to the markers of the international 10–10 system (Figure 2), and all probe sets were carefully inspected and adjusted to ensure consistent placement among the participants within each pair and across all pairs. Additionally, each child's head size was measured (*M* = 53 cm, SD = 1.22), and the appropriate headset size was selected based on these measurements.

### 2.4 Behavioral Measurements

#### 2.4.1 Child's Behavioral Problems

Mothers rated children's behavioral problems via externalizing and internalizing subscales of the Child Behavior Checklist/6‐18 (CBCL, Achenbach 2001). The CBCL has been widely used in China, demonstrating adequate reliability and validity (Wang and Liu 2018; Xing et al. 2011). The checklist consists of 113 items, each rated on a 3‐point scale: 0 = *not true of the child*, 1 = *somewhat or sometimes true*, and 2 = *very true/often*. The externalizing problems subscale includes 18 items related to aggressive behavior (e.g., “gets in many fights”) and 17 items related to rule‐breaking behavior (e.g., “breaks school rules”). The Internalizing problems subscale includes 13 items related to anxiety/depression (e.g., “cries a lot”) and eight items related to withdrawal/depression (e.g., “rather be alone”). In the present study, scores from 35 items were averaged to form a score for externalizing problems, and scores from 21 items were averaged to form a score for internalizing problems. In this study, the Cronbach's *α* values for externalizing and internalizing problems were acceptable (0.91 at T1, 0.86 at T2, and 0.85 at T3).

#### 2.4.2 Mother–Child Behavioral Synchrony

The coding of mother–child behavioral synchrony involved two types: simultaneous behavioral synchrony and temporally sequential behavioral synchrony. For simultaneous synchrony, we coded whether the mother and child were jointly attending to an external object. This included situations where both the parent and child focused on the same object (e.g., the Etch‐A‐Sketch or the desk) or engaged in a shared activity, such as drawing together (Harrist and Waugh 2002). For sequential synchrony, three steps were involved. First, two types of interactive behaviors were identified: (1) initiation—any action by one member of the dyad intended to elicit a response from the other; and (2) response—an action that directly follows and corresponds to a behavior initiated by the other member of the dyad. These behaviors were coded according to Lewis and Feiring's (1989) scheme. Second, two types of sequential synchronous behaviors were distinguished: MLCF synchrony and CLMF synchrony, depending on which member initiated the interaction (Feldman 2007; Markova et al. 2019). Then, the percentage of the total interaction duration occupied by each type of behavioral synchrony was used to quantify mother–child behavioral synchrony, just as in previous similar studies (Hoyniak et al. 2021; Piazza et al. 2020). In addition, we also calculated the average duration of each type of sequential synchronous interaction.

Four coders participated in thorough training sessions, which included an in‐depth review of the coding systems, clear explanations of the criteria, and hands‐on practice. Training continued until coders achieved a high level of agreement, with intraclass correlations (ICCs) of 0.75 or higher. After training, two coders independently reviewed the videos and reached a consensus on the final scores. The intercoder reliability demonstrated strong consistency across measures, with ICC values of 0.99 for simultaneous synchrony, 0.99 for MLCF synchrony, and 0.98 for CLMF synchrony, reflecting the robustness of the coding procedures.

### 2.5 Data Analysis

#### 2.5.1 Preprocessing of fNIRS Data

We used Homer, a MATLAB (The MathWorks Inc., Natick, MA, United States) toolbox, to convert and preprocess the fNIRS data. The optical density data were converted to HbO and HbR using the “hmrR_OD2Conc” function, based on the modified Beer–Lambert Law, with an age‐specific differential path length factor (Duncan et al. 1996). HbO concentrations were used in this study because they are a reliable marker of changes in regional cerebral blood flow and have a high signal‐to‐noise ratio (Hoshi 2007).

Before preprocessing, the first 30 s of baseline data and the first 10 s of data from the cooperative task were removed to exclude unstable periods from the fNIRS system. A quality check was performed using a 5‐s running‐window procedure (Zhao et al. 2021, 2022), which led to the exclusion of five mother–child pairs. Data preprocessing was carried out using functions from Homer 3 (Huppert et al. 2009). Specifically, motion artifacts were detected and corrected using a discrete wavelet transformation filter (Molavi and Dumont 2012). Principal component analysis was then applied to remove global physiological noise, such as skin blood flow, with a variance threshold set at 80% to ensure a lenient level of noise removal (Y. Zhang et al. 2005). The threshold of variance to be removed was set as 80% at a lenient level. Finally, a bandpass filter (0.01–0.7 Hz) was applied to eliminate high‐frequency and low‐frequency noise, which is suitable for natural interactions (Zhao et al. 2021).

#### 2.5.2 Mother–Child Neural Synchrony

**FIGURE 3:**
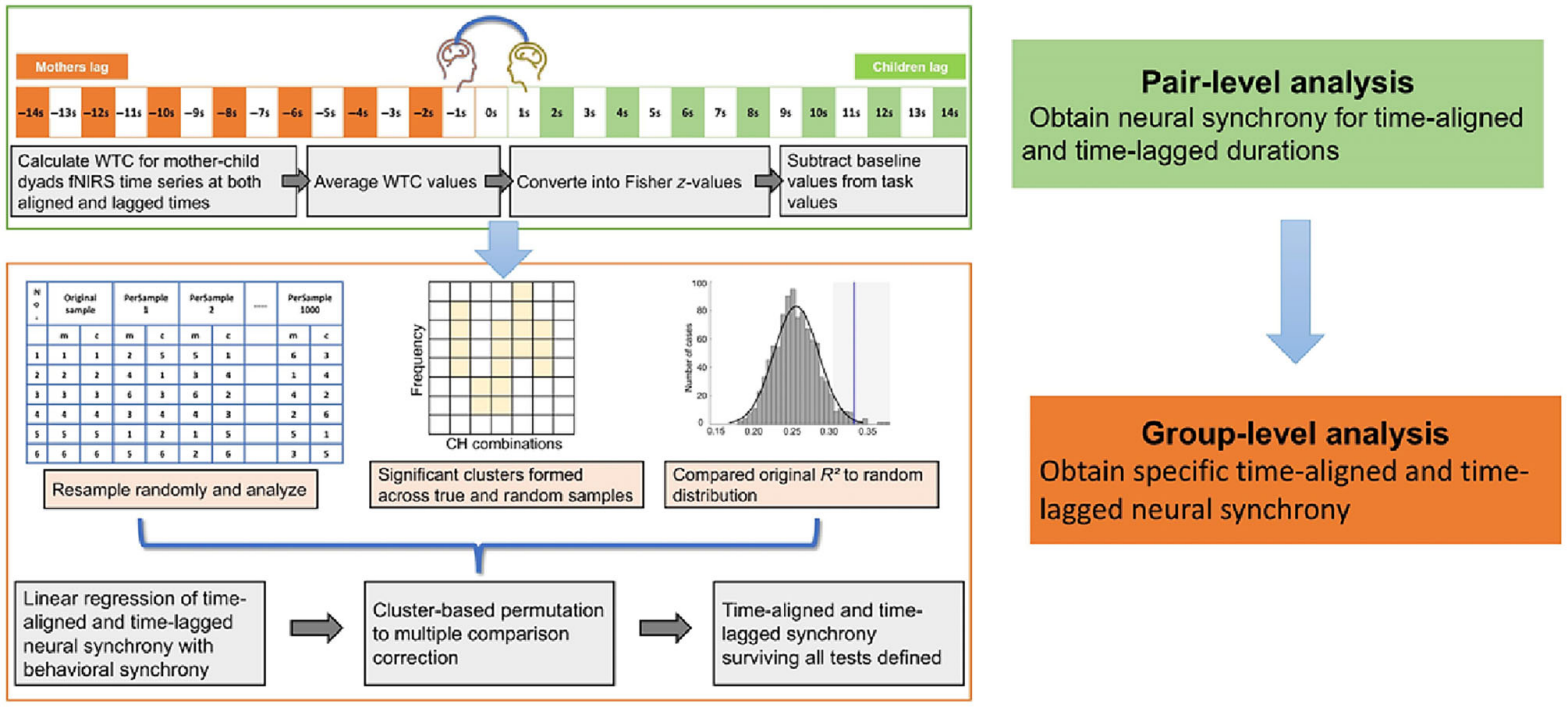
Analysis pipeline for neural synchrony.

To obtain neural synchrony (Figure 3), we first conducted pair‐level analyses, followed by group‐level analyses (Long et al. 2022). In the pair‐level analysis, both time‐aligned and time‐lagged neural synchrony across different lag durations between the two fNIRS time series, one for the mother and the other for the child. Time‐aligned neural synchrony was calculated by directly comparing the fNIRS time series of the mother and child at corresponding time points without introducing any temporal offsets. Time‐lagged neural synchrony was quantified by shifting the mother's time series forward or backward relative to the child's, in steps of 1 s from 1 to 14 s. These calculations were performed using the MATLAB function “wcoherence,” which computes WTC (Grinsted et al. 2004) as a function of frequency and time (Torrence and Compo 1998). A two‐dimensional matrix of coherence values was generated, with frequency represented by rows and time points by columns. Time‐aligned synchrony values were extracted from the matrix at zero lag within the target frequency band, while time‐lagged synchrony values were derived by recalculating coherence for each temporal shift. All 256 possible channel combinations between mothers and children were evaluated for each temporal shift. WTC values were averaged separately across baseline and task periods, followed by Fisher *z*‐transformations. Finally, the Fisher *z*‐value from the baseline was subtracted from that of the task period, yielding indices of time‐aligned neural synchrony and time‐lagged neural synchrony across different lag durations.

In the group‐level analyses, we obtained time‐aligned and time‐lagged neural synchrony following a similar approach to Zhao et al. (2021). Specifically, we employed linear regression to identify neural synchrony associated with two types of behavioral synchrony: simultaneous behavioral synchrony (e.g., parent and child behaviors co‐occurring within the same timeframe) and sequential behavioral synchrony (e.g., parent behavior preceding child behavior within a specified time window). These analyses were conducted for all 256 channel combinations, representing all pairwise combinations across predefined channels of interest. For time‐lagged analyses, we evaluated multiple lag durations, ranging from −14 to +14 s. To control for multiple comparisons arising from the large number of channel pairs and time‐lagged analyses, we employed a cluster‐based permutation approach with a significance threshold of *p* < 0.05 (Maris and Oostenveld 2007; Zhao et al. 2021). Participants were randomly reassigned into new pairs, and time‐aligned as well as time‐lagged neural synchrony were recalculated for each permutation. Clusters of significant effects were formed along neighboring significant frequency bands to minimize confounding from global physiological noise. For each cluster, we averaged the *R* ^2^ values within the largest cluster, defined as the cluster with the highest sum of significant values, based on its statistical robustness. This process was repeated 1000 times to generate a distribution of cluster‐based *R* ^2^ values. Finally, we compared the original *R* ^2^ values to this distribution to assess significance (*p* < 0.05). Time‐aligned neural synchrony (with no lag) and time‐lagged neural synchrony (across multiple lag durations) that passed all significance tests were considered specific neural synchrony.

#### 2.5.3 Relations Between Mother–Child Synchrony and Behavioral Problems

Comparisons between the duration of CLMF synchrony and the specific lag of time‐lagged neural synchrony, as well as between the duration of MLCF synchrony and the specific lag of time‐lagged neural synchrony, were performed using one‐sample *t*‐tests.

Relations between mother–child synchrony and behavioral problems were examined through several analyses. First, we conducted descriptive statistics and bivariate correlations among the study variables. Second, due to the relatively small sample size, we developed two separate path analysis models to examine the relations between behavioral synchrony and externalizing problems, and between behavioral synchrony and internalizing problems, across three different time points. Additionally, two separate path analysis models were used to examine the relations between neural synchrony and two types of behavioral problems. To assess the long‐term predictive power of behavioral synchrony, we conducted sensitivity analyses. Each path analysis model controlled for a specific behavioral problem from the previous time point, and both direct and indirect effects were examined. All these models were analyzed using Mplus 8.0 (Muthén and Muthén 2012), and the significance of the paths was determined via bootstrapping using 1000 random samples to estimate the effects and their 95% confidence intervals (CIs). Missing data were handled using the full information maximum likelihood method (Schafer and Graham 2002). Child gender was not included as a control variable, as no statistically significant differences were found in behavioral problems based on gender. It should be noted that these SEMs are exploratory; the ratio of participants to parameters does not provide sufficient statistical power for definitive conclusions. However, our analyses may generate grounded hypotheses for further research in larger samples.

## 3 Results

### 3.1 Mother–Child Neural Synchrony and Its Relation to Behavioral Synchrony

No significant time‐aligned neural synchrony was found to be associated with the percentage of simultaneous behavioral synchrony (*p* > 0.05).

**FIGURE 4:**
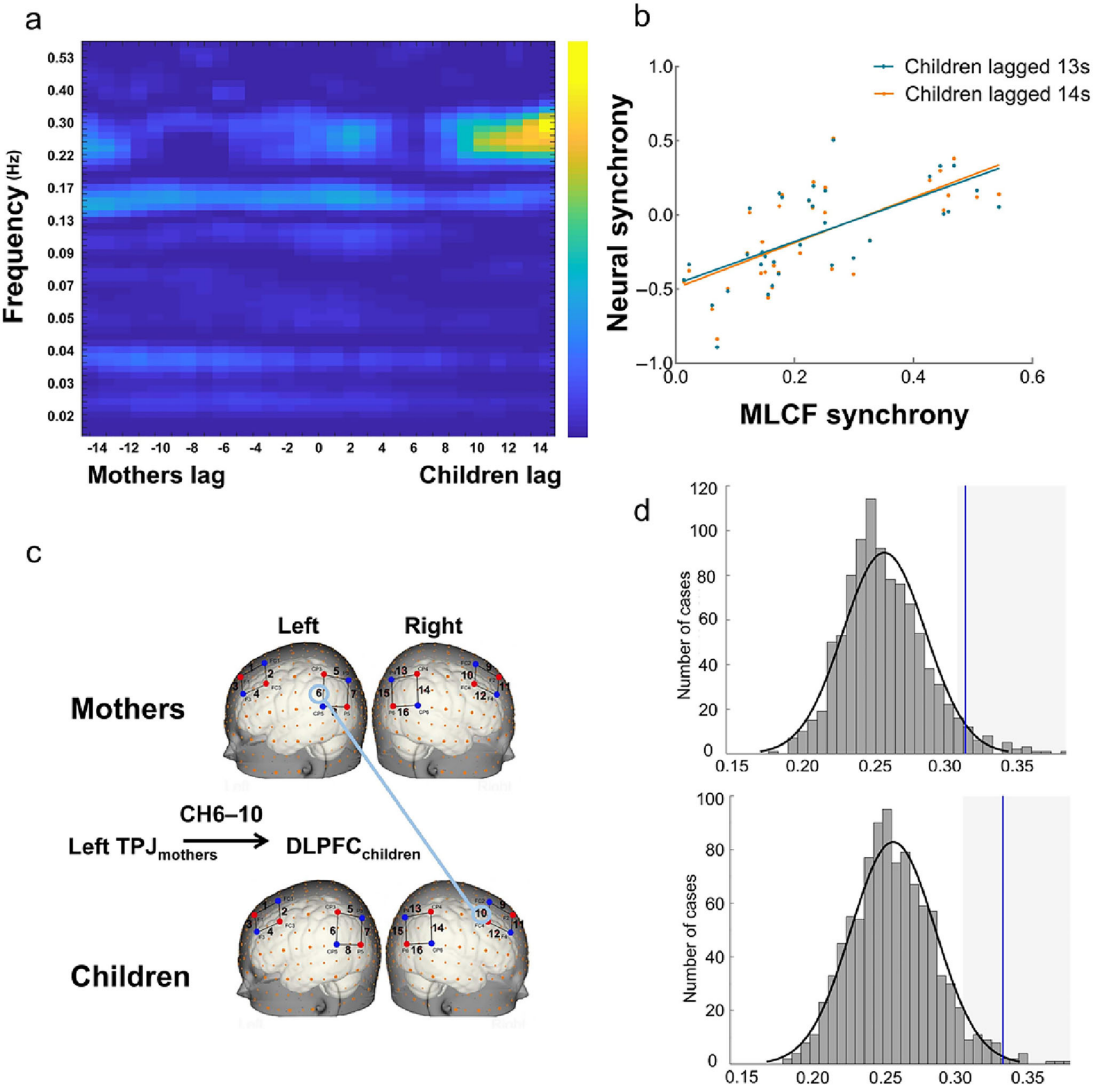
Neural synchrony with children's brain activity lagged 13–14 s associated with MLCF synchrony. (a) The regression results of MLCF synchrony and neural synchrony with children's brain activity lagged 13 s at left TPJ_mother_ → dlPFC_child_. The *y*‐axis represents the frequency in Hz (0.02–0.6 Hz), and the *x*‐axis represents the time lags from −14 s (the mothers’ brain activity lagged behind that of the children) to 14 s (the children's brain activity lagged behind that of the mothers) with a step size of 1 s. The position of 0 represents time‐aligned neural synchrony. The colored bar indicates the value of *R* ^2^. (b) Cluster‐based permutation approach to the correction of multiple comparisons. The gray areas indicate significance within the 5% area (gray color) (corresponding to a significance level of 0.05, one‐tailed) of the distribution generated by the permutation test. The blue lines indicate the position of the original *R* ^2^ within 0.2–0.3 Hz when the children's brain activity lagged behind that of the mothers by 13–14 s. (c) The anatomical positions of the regression results. (d) The regression results of MLCF synchrony and neural synchrony with children's brain activity lagged 13–14 s. Scatter plots of the associations between MLCF synchrony and neural synchrony with children's brain activity lagged 13–14 s.

However, the results on neural synchrony showed that when the children's brain activity lagged behind that of the mothers by 13–14 s, there was a significant positive correlation between the percentage of MLCF synchrony and neural synchrony at a frequency range of 0.2–0.3 Hz (Figure 4a,b, *R* ^2^_13 s_ = 0.32, *R* ^2^_14 s_ = 0.34, *p* < 0.001). The correlation was found at the left TPJ (CH6) of the mothers and the dlPFC (CH10) of the children (Figure 4c). The *R* ^2^ distribution generated from the random‐pairing permutation procedure was shown in Figure 4d and confirmed the significant association between the percentage of MLCF synchrony and the children's lagged neural synchrony at CH6–10 within 0.2–0.3 Hz after correction for multiple comparisons (*p* < 0.05).

No significant time‐lagged neural synchrony was found to be associated with the percentage of CLMF synchrony (*p* > 0.05). Therefore, subsequent analyses focus solely on the hypothesis concerning 13‐ to 14‐s time‐lagged neural synchrony associated with MLCF synchrony.

Next, to test the relationship between behavioral duration and neural time‐lag, we compared the average time‐lag of MLCF synchrony (*M* = 11.61, SD = 6.67) with fixed neural synchrony time‐lags of 13 s and 14 s using one‐sample *t*‐tests. The results indicated no significant difference between the MLCF average time‐lag and 13 s, *t*(32) = −1.20, *p* = 0.24. However, a significant difference was found when comparing with 14 s, *t*(32) = −2.06, *p* = 0.048.

Furthermore, the direction of the time‐lagged neural synchrony was from the leader to the follower, specifically from the mother to the child. Based on these findings, subsequent analyses of neural synchrony were focused exclusively on instances where the children's brain activity lagged behind that of the mothers by 13 s.

### 3.2 Descriptive Statistics and Correlation Between Mother–Child Synchrony and Children's Behavioral Problems

**FIGURE 5:**
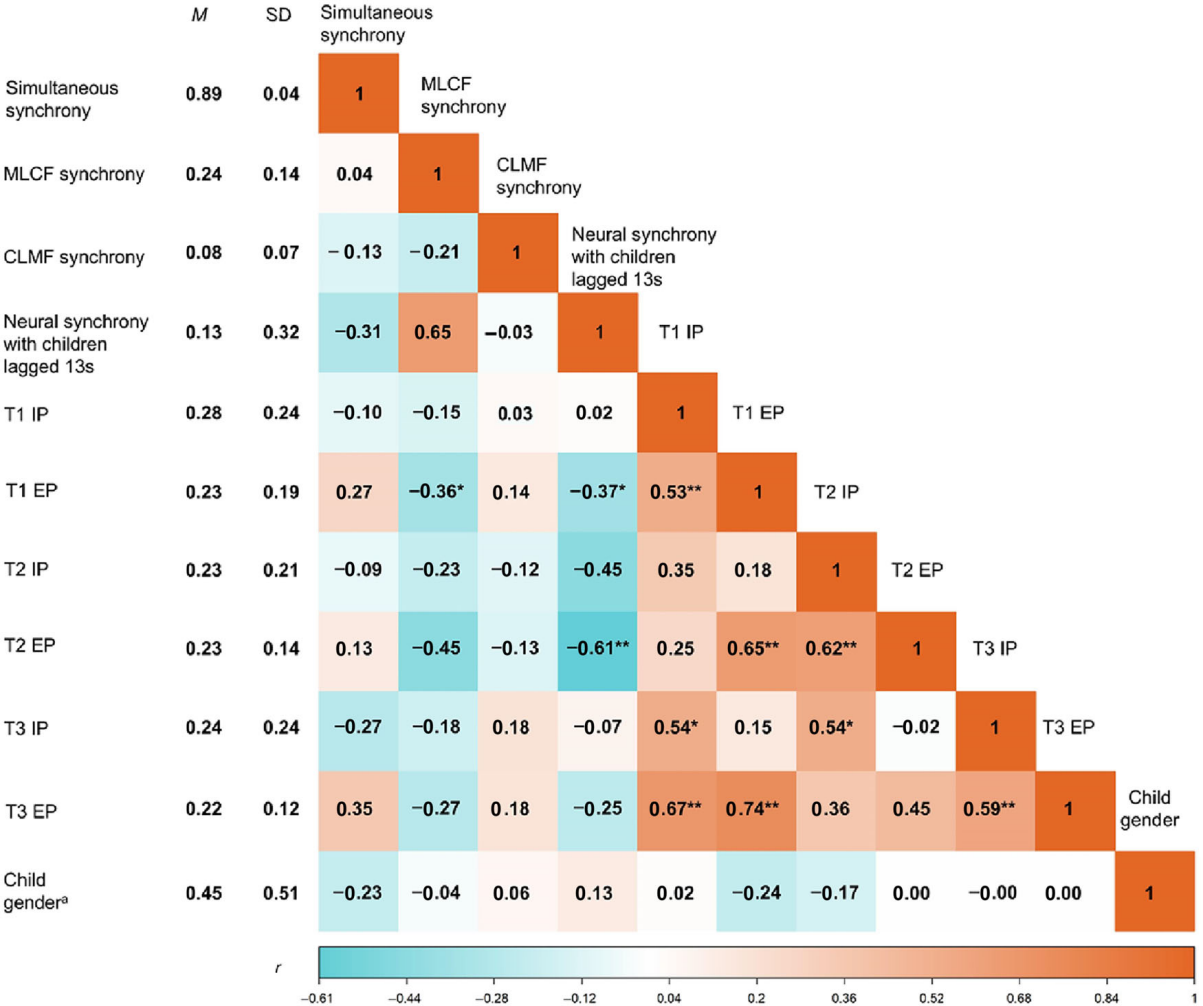
Means (*M*), standard deviations (SD), and correlations among synchronous behaviors, behavioral problems, and child gender. ^a^boy = 0, girl = 1. *p < 0.05, **p < 0.01, ***p < 0.001. Behavioral synchrony: simultaneous synchrony; CLMF synchrony = child‐led mother‐following synchrony; EP = externalizing problems; IP = internalizing problems; MLCF synchrony = mother‐led child‐following synchrony; Neural synchrony with children lagged 13 s = neural synchrony with children's brain activity lagged 13 s; T1 = age 7; T2 = age 9; T3 = age 11. The above notes also apply to the figure below.

The descriptive statistics and correlations for all studied variables are presented in Figure 5. The percentages of MLCF synchrony and CLMF synchrony were compared, and the results of a *t*‐test showed that the percentage of two sequential synchronous behaviors differed significantly, *t* (32) = 5.32, *p* < 0.001. Specifically, the percentage of MLCF synchrony is more than that of CLMF synchrony.

We recoded children's gender as a binary variable (boy = 0, girl = 1) to examine its correlation with other variables. No significant relations were found between gender and behavioral problems outcomes (Figure 5, *p* > 0.05). Significant within‐domain stability over time was observed, with correlations ranging from 0.45 to 0.74 (Figure 5, *p* < 0.01 to *p* < 0.1), except for the correlation of internalizing problems between T1 and T2 that was 0.35 (Figure 5, *p* = 0.19). Within‐time correlations between externalizing and internalizing problems demonstrated strong co‐development of behavioral problems at T1 (*r* = 0.53, *p* = 0.002), T2 (*r* = 0.62, *p* = 0.008), and T3 (*r* = 0.58, *p* = 0.007).

The correlations revealed a negative association between higher levels of MLCF synchrony and fewer externalizing problems at T1 (Figure 5, *r* = −0.36, *p* = 0.048). Additionally, 13‐s lagged neural synchrony showed a marginally significant negative association with externalizing problems at T1 (*r* = −0.34, *p* = 0.06) and a significant negative association at T2 (*r* = −0.56, *p* = 0.02). No significant association was found at T3. No significant association was found between simultaneous synchrony and behavioral problems.

### 3.3 Mother–Child Synchrony Predicts Behavioral Problems Over Three Time Points

**FIGURE 6:**
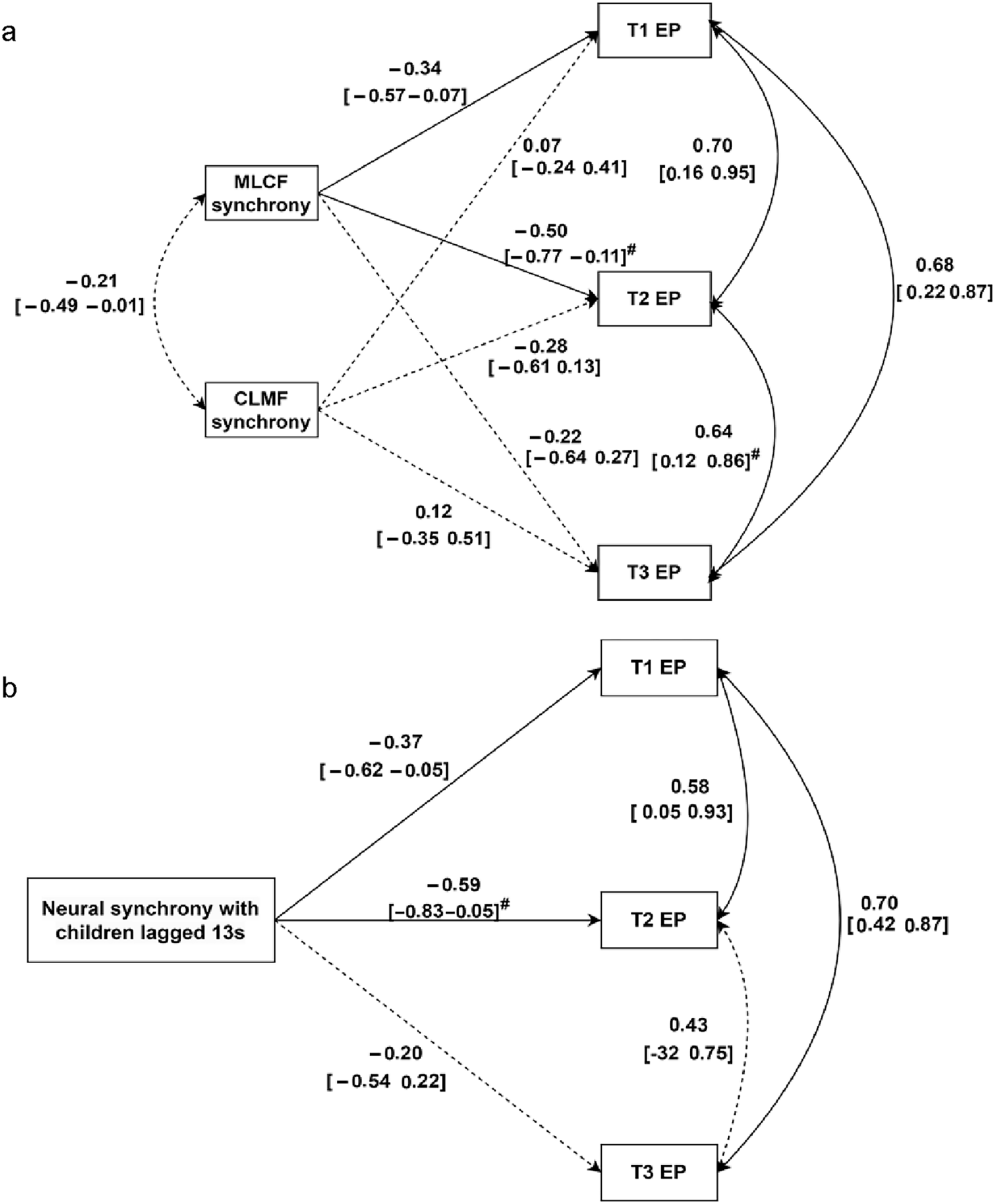
Path analysis models for behavioral synchrony (MLCF synchrony and CLMF synchrony), neural synchrony (13‐s lagged neural synchrony), and externalizing problems at T1, T2, and T3, respectively (*N* = 33). Solid lines indicate significant paths, and dashed lines represent nonsignificant paths. The values on the paths represent path coefficients and confidence intervals (CI). **^#^** represents 90% CI. The above notes also apply to the figure below.

The results of two path models examining the relations between behavioral synchrony, 13‐s lagged neural synchrony, and externalizing and internalizing problems across three time points indicated that both MLCF synchrony and 13‐s lagged neural synchrony were significant predictors of externalizing problems, but not internalizing problems. All these models were fully saturated (*χ* ^2^ = 0.00, df = 0.00). Specifically, as shown in Figure 6a, higher MLCF synchrony was associated with fewer externalizing problems at T1 (*β* = −0.34, 95% CI [−0.57, −0.07]) and T2 (*β* = −0.50, 90% CI [−0.77, −0.11]), but this effect did not persist at T3 (*β* = −0.22, 95% CI [−0.64, 0.27]). No significant paths were found between CLMF synchrony and externalizing problems at any time point. Similarly, as shown in Figure 6b, 13‐s lagged neural synchrony was linked to fewer externalizing problems at T1 (*β* = −0.37, 95% CI [−0.62, −0.05]) and T2 *(β* = −0.59, 90% CI [−0.83, −0.05]), but this association was not significant at T3 (*β* = −0.20, 95% CI [−0.54, 0.22]).

### 3.4 Sensitivity Analysis

**FIGURE 7:**
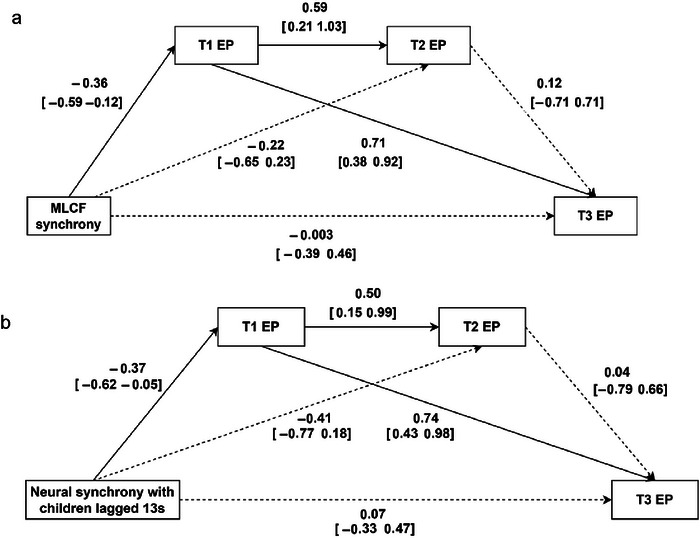
Path analysis models for behavioral synchrony (MLCF synchrony), neural synchrony (13‐s lagged neural synchrony), and externalizing problems at T1, T2, and T3 (*N* = 33).

To further examine the associations between mother–child synchrony and externalizing problems, we conducted sensitivity analyses for both MLCF synchrony and 13‐s lagged neural synchrony. The path models for both synchrony measures, presented in Figure 7a,b, were fully saturated (*χ* ^2^ = 0.00, df = 0.00). In both models, synchrony significantly predicted externalizing problems only at Time 1 (T1), with MLCF synchrony and 13‐s lagged neural synchrony showing a similar effect (MLCF synchrony: *β* = −0.36, 95% CI [−0.59, −0.12]; 13‐s lagged neural synchrony: *β* = −0.37, 95% CI [−0.62, −0.05]).

Two significant indirect effects were identified in each model. For MLCF synchrony, the first indirect effect showed an association with externalizing problems at T1, which in turn predicted externalizing problems at T2, *B* = −0.031, 95% CI [−0.124, −0.003]. The second indirect effect indicated that MLCF synchrony was associated with externalizing problems at T1, which subsequently predicted externalizing problems at T3, *B* = −0.027, 95% CI [−0.060, −0.010].

Similarly, in the 13‐s lagged neural synchrony model, two significant indirect effects were identified. The first indirect effect indicated that neural synchrony was associated with externalizing problems at T1, which in turn predicted externalizing problems at T2, *B* = −0.085, 95% CI [−0.377, −0.008]. The second indirect effect showed that neural synchrony was associated with externalizing problems at T1, which subsequently predicted externalizing problems at T3, *B* = −0.091, 95% CI [−0.226, −0.019].

These results indicated that both MLCF synchrony and 13‐s lagged neural synchrony were associated with externalizing problems at the initial time point (T1), and these associations might continue across subsequent time points through indirect pathways.

## 4 Discussion

In the present study, we first investigated the relations between behavioral synchrony and neural synchrony during cooperative drawing interactions when children were seven years old. Specifically, we examined how both behavioral and neural synchrony between mother and child predicted behavioral problems from middle childhood to preadolescence. Our hypothesis, which proposed that both behavioral and neural synchrony would be associated with fewer behavioral problems, was partially supported. We found that 13‐s lagged neural synchrony was directed from the mother's left TPJ to the child's dorsolateral prefrontal cortex (dlPFC), with the lag duration corresponding to the average duration of MLCF synchrony. Furthermore, both higher levels of MLCF synchrony and 13‐s lagged neural synchrony predicted fewer externalizing problems across the transition from middle childhood to preadolescence.

### 4.1 The Neural Basis of Behavioral Synchrony in Mother–Child Cooperative Interactions

In this study, we observed that during cooperative drawing interactions between 7‐year‐old children and their mothers, temporally sequential behavioral synchrony predominantly took the form of MLCF synchrony. This suggests that mothers often take a guiding role, regulating their children's behavior, which is consistent with previous research showing that mothers provide cognitive scaffolding to support children's learning (Gauvain and Perez 2008). The prevalence of MLCF synchrony may also reflect the strong caregiving roles of mothers in China, characterized by a sense of responsibility and active engagement, often conceptualized as *guan* (Lan et al. 2019). Children, in turn, are highly responsive to their mothers’ cues, adjusting their actions to maintain synchrony, aligning with cultural practices of filial piety (Wang et al. 2022). Importantly, we found that the neural synchrony associated with MLCF synchrony followed a time‐lagged pattern, with the child's brain activity lagging behind the mother's by 13 s. The emergence of a specific time lag is consistent with a growing body of hyperscanning research showing that time‐lagged neural synchrony reflects predictive and regulatory processes unfolding over behaviorally meaningful temporal scales, rather than instantaneous coupling (Jiang et al. 2021). This neural synchrony likely reflects shared social cognitive and attentional processes, such as mutual correlation and adaptation (Hamilton 2021), as well as the communication of social signals (Leong et al. 2017; Wass et al. 2018). Empirically, similar multi‐second lag durations have been reported across interactive contexts, including teacher–student instruction (Zheng et al. 2018) and mother–child shared book reading (Zhai et al. 2023), where they align with the duration of interactional turns. Taken together, these findings suggest that the lag observed in the present study may reflect a temporally structured leader–follower dynamic through which maternal guidance is neurally integrated into the child's ongoing behavioral regulation during cooperation.

In contrast to time‐lagged neural synchrony, we did not find significant associations between simultaneous behavioral synchrony and time‐aligned neural synchrony. One possible explanation is that, compared to young children, school‐aged children show significant improvements in attention and executive function (Klenberg et al. 2001). As a result, simultaneous behavioral synchrony, such as joint attention in this case, may become less sensitive to these developmental changes at this age. Moreover, future studies could further explore the role of other cognitive or social factors that may contribute to this shift, such as changes in social motivations or cognitive control, which could influence the relationship between behavioral and neural synchrony at different developmental stages.

### 4.2 Mother–Child Synchrony and Behavioral Problems

Consistent with previous research, higher levels of behavioral synchrony between mothers and children have been linked to fewer behavioral problems (Bureau et al. 2021; Im‐Bolter et al. 2015; Pasiak and Menna 2015). The current study extends this understanding, prospectively examining this relation in a Chinese cultural context. As mentioned in the previous section, during the cooperative drawing task, MLCF synchrony was the predominant form of temporally sequential behavioral synchrony. Our results further showed that MLCF synchrony was negatively related to externalizing problems in Chinese children, which is in line with earlier studies. For example, Gao et al. (2015) found that parental *guan* was associated with lower levels of proactive and reactive aggression in Hong Kong youth. When the mother initiates appropriate behaviors and the child demonstrates increased compliance (Lan et al. 2019), it suggests the child is adapting to and understanding the mother's role and instructions. Effective behavioral management, such as consistent monitoring and setting boundaries, can mitigate externalizing problems by improving children's self‐regulation and compliance (McKee et al. 2008; Pinquart 2017; Rothenberg et al. 2020).

Similar to behavioral synchrony, our results indicate that 13‐s lagged neural synchrony between mothers and children is negatively associated with externalizing problems. These findings support Feldman's biobehavioral synchrony model (Feldman 2012a), highlighting the significance of time‐lagged parent–child neural synchrony. From a developmental perspective, this observed lag may reflect the time scale over which maternal guidance and socioemotional cues are processed within the interaction. Externalizing behaviors are closely linked to deficits in inhibitory control and emotion regulation, processes that may rely on sustained integration of social input over time rather than solely on immediate reactivity (Chaku et al. 2024). Within this framework, time‐lagged neural synchrony unfolding over this behavioral timescale may be associated with patterns of parental scaffolding that support children's emerging self‐regulatory capacities.

Specifically, the time‐lagged neural synchrony observed between the mother's left TPJ and the child's dlPFC reflects the roles these regions play in social cognition and cooperation (Schilbach and Redcay 2024). The TPJ, involved in monitoring socioemotional significance and representing mental states (Hoehl et al. 2021; Samson et al. 2004; Saxe and Wexler 2005), suggests that the mother anticipates and aligns her actions with the child's mental state (Kochanska and Aksan 2004), fostering neural synchrony (Nguyen et al. 2020). The child's delayed activation in the dlPFC—a region linked to cognitive control and emotion regulation (Balconi and Pagani 2015; Gvirts and Perlmutter 2020; Zhang et al. 2024)—indicates their effort to manage emotions and follow the mother's guidance during cooperative tasks, such as drawing together. This neural alignment may capture the child's developing capacity for emotional regulation and shared intentionality, which is critical in reducing externalizing problems. Additionally, time‐lagged neural synchrony underscores the mother's role in guiding and regulating interactions, consistent with the concept of *guan*. This guiding role not only fosters immediate compliance but also extends to the child's behavior in unfamiliar social contexts (Zhao et al. 2021), further supporting the negative association between neural synchrony and externalizing problems.

Unlike the findings of Quiñones‐Camacho et al. (2022), the negative relationships between the lead‐lag pattern of neural synchrony and internalizing problems were not significant in this study. Parents may find it challenging to evaluate internalizing symptoms in children, as these issues are often experienced privately and subjectively (Hinshaw et al. 1992). This fundamental difficulty could account for the generally weaker associations observed within the internalizing problems in this study. This discrepancy may be partly due to the younger age of the children in Quiñones‐Camacho et al. (2022), who focused on preschool‐aged children, where internalizing problems are often more readily identifiable. In contrast, the internalizing problems observed in the school‐aged children in our study may be more subtle and less overt, making them harder to detect by their mothers. On the other hand, Quiñones‐Camacho et al. (2022) employed a frustrating task to elicit distressing emotions in children during parent–child interactions, whereas our study examined interactions within a play task context. It is possible that neural synchrony may predict different types of problem behaviors depending on the emotional state of the child, highlighting the need for future research to explore these dynamics further.

### 4.3 Limitations and Future Directions

As this is a preliminary study, it is important to acknowledge some limitations of the current study. First, the generalizability of the findings may be limited due to the small and selective sample, which primarily included highly educated, middle‐ to high‐income families from a large metropolitan area. Second, the limited sample size in this study prevented the use of methods like Latent Growth Curve modeling to explore the complex relationship between behavioral and neural synchrony and changes in children's behavioral problems. Accordingly, the present findings should be interpreted as preliminary and hypothesis‐generating rather than confirmatory. Future research should aim to include larger and more diverse samples to enable such advanced analytical techniques and provide a more nuanced understanding of these dynamics. Third, the behavioral problems were only reported by mothers. This reliance on a single informant may introduce bias and limit the accuracy of the measurements. Future research should consider including multi‐informant reports, such as fathers, teachers, and the children themselves (De Los Reyes et al. 2015). This would provide a more comprehensive assessment of behavioral problems and enhance the validity and reliability of the findings. Moreover, because the present study focused on Chinese mother–child dyads, the observed mother‐led synchrony may reflect culturally normative parenting practices and may not generalize to contexts in which child‐led parenting is more common. Future cross‐cultural research is needed to determine whether these patterns are culture‐specific or more universal.

## 5 Conclusions

In conclusion, this study advances our understanding of the relationship between behavioral and neural synchrony in mother–child interactions at middle childhood. It also addresses an important gap in predicting behavioral problems through preadolescence. Specifically, time‐lagged neural synchrony was associated with the predominant form of behavioral synchrony, involving the mother's TPJ and the child's dlPFC. Furthermore, both MLCF behavioral synchrony and 13‐s lagged neural synchrony were linked to fewer externalizing problems in children. These findings shed new light on the neurocognitive processes underpinning mother–child interactions, contributing to a deeper understanding of how these interactions support children's social‐emotional development from middle childhood to preadolescence.

## Author Contributions

**Chao Liu**: conceptualization, writing – review & editing, writing – original draft, methodology, validation, visualization, data curation, software, investigation, and formal analysis. **Xi Liang**: conceptualization, methodology, supervision, data curation, writing – review and editing. **Xiaoxu Meng**: investigation, data curation, visualization. **Nanhua Cheng**: funding acquisition, resources, visualization. **Shan Lu**: investigation, data curation, visualization. **Liu Bai**: formal analysis, visualization. **Bingbing Song**: investigation, data curation. **Chunming Lu**: funding acquisition, supervision, writing – review and editing. **Zhengyan Wang**: conceptualization, funding acquisition, supervision, resources.

## Funding

This work was supported by the National Natural Science Foundation of China (32471119, 62293550, 62293551, 32300896).

## Ethics Statement

At the end of each visit, children were given a token of appreciation for their participation. The study protocol was approved by the Psychological Ethics Committee of Capital Normal University and the Institutional Review Board of the State Key Laboratory of Cognitive Neuroscience and Learning at Beijing Normal University. All procedures involving human participants were conducted in accordance with the Ethical Principles of the Chinese Psychological Society.

## Consent

Written informed consent was obtained from the mothers on behalf of themselves and their children.

## Conflicts of Interest

The authors declare no conflicts of interest.

## Data Availability

The data and materials used in this study are available upon request from the corresponding author.
